# Lonesome No More: The Associative Thalamus and the Feeling of Being Alone

**DOI:** 10.1016/j.bpsgos.2026.100808

**Published:** 2026-09-17

**Authors:** James M. Shine

**Affiliations:** School of Medical Sciences, The University of Sydney, Sydney, New South Wales, Australia

The thalamus has been slow to shed its reputation as a relay. To date, it is still introduced by many as a passive way station where sensory signals pause on their way to the cerebral cortex. However, only a minority of thalamic neurons carry peripheral information at all, and many thalamic cells are not even constructed in a way that would allow them to act as effective relays. Most of the thalamic neurons are actually driven by the cerebral cortex itself or by nonsensory subcortical structures, and their function is to route, gate, sustain, and coordinate activity between cortical regions rather than to report on the world outside. In a recent study published in *Biological Psychiatry: Global Open Science*, Mäki-Marttunen *et al.* (1) add an unexpected entry to the list of things this associative machinery appears to track: how alone a person feels.

Working on a dataset of close to 50,000 participants from the UK Biobank, the authors divided the thalamus into its constituent nuclei and asked which of them, if any, varied with the subjective report of loneliness. The answer was regionally specific. The volume reductions were not spread evenly throughout the structure but were gathered in its higher-order territory—the mediodorsal, pulvinar, and lateral posterior nuclei—which are the regions that have the least to do with relaying sensation and the most to do with the associative machinations of the rest of the brain. The whole-thalamus association that earlier work had reported (2) turns out, at this resolution, to be carried by particular subsections of this fascinating neural hub.

The finding that stays with me is not necessarily the anatomy itself, but what the anatomy is tracking. When the authors set the subjective experience of loneliness against the objective fact of social isolation, the thalamic signal followed the feeling. Participants who felt lonely despite regular social contact had the smallest thalami; those who were isolated but untroubled by it were indistinguishable from the contented majority. Whatever the thalamus is registering here, it tracks something other than the sheer quantity of a person’s social contacts.

A structural finding of this kind invites a mechanistic reading, and here the higher-order thalamic nuclei earn their place. The authors themselves note the relevant circuitry in their discussion: the pulvinar’s role in directing cortical attention, the mediodorsal nucleus’s dense reciprocal traffic with the prefrontal cortex, and the possibility that these contribute to the vigilance for social threat that has long been described in lonely people (3). I want to take that intuition one step further because the level at which these nuclei matter is not, I think, the individual structure but the broader context to which each nucleus belongs.

Consider what the 2 implicated nuclei are known to do. The pulvinar adjusts the transmission of information between cortical areas according to the demands of attention (4); the mediodorsal nucleus amplifies and sustains the prefrontal activity that holds a specific task in place (5). Neither is relaying content, so much as constraining how the cortex processes it. The loneliness phenotype then may have less to do with any single nucleus being smaller and more to do with how the thalamocortical loop weights incoming social information: a form of gain control exercised over cognition rather than over sensation (6,7). The distinction matters because gain of this kind shapes the landscape over which cortical activity settles, biasing which interpretations are easy to reach and which are not. A structure that constrains what the cortex attends to and how strongly it commits to a given reading is well placed to tilt a person toward construing ambiguous social signals as unwelcoming.

That framing also makes sense of the dissociation between feeling and circumstance. Loneliness is, by definition, a discrepancy: the gap between the connections a person has and the connections that they want. A circuit that compares what the cortex predicts about the social world with what actually arrives is a natural place for such a discrepancy to be computed, as well as miscomputed (8). The smaller thalamus of the lonely but-not-isolated participant would then indicate not how little company they keep, but how their expectations of connection are being reconciled with what they actually encounter. This fits the shape of the authors’ own comparison: it is the group that felt lonely despite company, and not the group that was isolated without distress, whose thalami were smaller.

None of this is settled by volume, and it is worth being clear about what the measure can and cannot do. The effects are small, as effects at this sample size tend to be, and cross-sectional volumetry offers a static shadow of a process that is inherently dynamic. For this reason, the authors’ own longitudinal analysis, which found no change in thalamic volume as loneliness came and went, is better read as a limitation of the instrument than as evidence against a thalamic contribution; 2 years of volume change is a coarse probe of a circuit whose relevant activity unfolds over milliseconds. What the finding calls for now is a functional study rather than a larger volumetric one, asking whether the thalamocortical coordination that these nuclei support is itself altered in lonely people, and whether that alteration precedes the feeling or follows it.

I hold a prior commitment to the view that the thalamus organizes cortical dynamics and does not merely serve them (9), and the reader should weigh what follows against it. What recommends this study is that it did not set out to test that view, and yet it arrived at it all the same. Asked a plain epidemiological question about loneliness, the thalamus answered from its associative nuclei, and it answered to a feeling rather than to a circumstance. The same higher-order territory is now implicated wherever the field examines closely how subcortical structures shape perception and awareness (10). Loneliness, which Kurt Vonnegut was content to call a disease and which has proven stubbornly resistant to explanation at the level of the cortex alone, may be one more phenomenon whose more fine-grained account is better understood when connected through the thalamus.
